# Hormesis in Plants: The Role of Oxidative Stress, Auxins and Photosynthesis in Corn Treated with Cd or Pb

**DOI:** 10.3390/ijms21062099

**Published:** 2020-03-19

**Authors:** Eugeniusz Małkowski, Krzysztof Sitko, Michał Szopiński, Żaneta Gieroń, Marta Pogrzeba, Hazem M. Kalaji, Paulina Zieleźnik-Rusinowska

**Affiliations:** 1 Plant Ecophysiology Team, Faculty of Natural Sciences, Institute of Biology, Biotechnology and Environmental Protection, University of Silesia in Katowice, 40-032 Katowice, Poland; mszopinski@us.edu.pl (M.S.); zgieron@us.edu.pl (Ż.G.); pzieleznik@us.edu.pl (P.Z.-R.); 2 Institute for Ecology of Industrial Areas, 40-844 Katowice, Poland; m.pogrzeba@ietu.pl; 3 Department of Plant Physiology, Institute of Biology, Warsaw University of Life Sciences WULS-SGGW, 02-776 Warsaw, Poland; hazem@kalaji.pl

**Keywords:** hormesis, growth, photosynthesis, chlorophyll *a* fluorescence, cadmium, lead

## Abstract

Hormesis, which describes the stimulatory effect of low doses of toxic substances on growth, is a well-known phenomenon in the plant and animal kingdoms. However, the mechanisms that are involved in this phenomenon are still poorly understood. We performed preliminary studies on corn coleoptile sections, which showed a positive correlation between the stimulation of growth by Cd or Pb and an increase in the auxin and H_2_O_2_ content in the coleoptile sections. Subsequently, we grew corn seedlings in hydroponic culture and tested a wide range of Cd or Pb concentrations in order to determine hormetic growth stimulation. In these seedlings the gas exchange and the chlorophyll *a* fluorescence, as well as the content of chlorophyll, flavonol, auxin and hydrogen peroxide, were measured. We found that during the hormetic stimulation of growth, the response of the photosynthetic apparatus to Cd and Pb differed significantly. While the application of Cd mostly caused a decrease in various photosynthetic parameters, the application of Pb stimulated some of them. Nevertheless, we discovered that the common features of the hormetic stimulation of shoot growth by heavy metals are an increase in the auxin and flavonol content and the maintenance of hydrogen peroxide at the same level as the control plants.

## 1. Introduction

Hormesis is a phenomenon that is defined as the stimulatory effect of low doses of toxic substances, e.g., heavy metals, on a single biological parameter of a given organism [1]. The hormetic effect is described by a reversed U-shaped biphasic curve in which a low dose of a toxic substance has a stimulatory effect; however, when the dose is increased, the toxic effect starts to be visible [2,3,4]. Hormesis is considered to be a universal phenomenon that is common in nature, which is independent of the type of stressor, the organism in which it occurs or the physiological process [2,3,4]. This notion is supported by numerous studies that have been carried out on a wide range of organisms (from microorganisms through plants to mammals) [5,6]. Some studies have suggested that hormesis represents an evolutionary adaptive response to environmental factors that interfere with homeostasis [7,8].

Growth, oxidative stress and/or photosynthetic activity are the physiological parameters that are most frequently examined by scientists who are interested in hormesis in plants [3,4,8,9,10,11]. Many scientists have connected the stimulation of plant growth during hormesis with a low level of oxidative stress [3,7,8,10,12]. Their results show that reactive oxygen species (ROS) are undoubtedly involved in the hormetic effect. However, there is a dearth of information that could explain the mechanisms that underlie the hormesis phenomenon [6].

Cd and Pb are heavy metals that are commonly known for their lack of any biological functions in plants [13] and for their toxic effects on plant growth and development [14,15,16,17]. Pb is mostly accumulated in roots, which negatively affects their growth and cell division. This metal is characterized by a low translocation from the root to shoot [18]. As a result, the shoot growth is inhibited to a lesser degree than that of the root [14,19,20]. By contrast, Cd is easily transported from the root to the aboveground parts of a plant and thereby exerts a negative influence on the whole plant [15,19]. Both metals cause a disturbance in the proper functioning of the root, which negatively affects the mineral nutrition of plants [14,15,21]. Moreover, Cd and Pb induce oxidative stress in plants and also affect photosynthesis, water relations and hormonal balance [15,21,22].

Coleoptile sections of the Poaceae family, which are excised from etiolated seedlings, are a model object in plant elongation growth studies because their cells do not undergo division but only elongate [23,24,25]. Coleoptile sections are frequently used to determine the influence of various factors on the elongation growth, e.g., heavy metals [26,27,28,29]. It was found that the Pb or Cd that was added to an incubation solution inhibited both the endogenous and exogenous auxin-induced growth of coleoptile sections [27,28,29]. On the other hand, Małkowski et al. [20] observed that the elongation growth of coleoptile sections that had been excised from corn seedlings growing for 24 h in the presence of Pb at 100 or 1000 μM, and then incubated in a control medium with auxin (IAA), was 47% or 69% higher, respectively, compared to the growth of the sections that had been cut from seedlings that had not been treated with the heavy metal. This phenomenon seems to be similar to hormetic stimulation of growth, although its mechanism is not known.

The growth of coleoptile sections is induced by indole-3-acetic acid (IAA). This applies to both endogenous growth and growth that is stimulated by exogenous auxin [25,30,31]. Schopfer et al. [32] documented that the presence of oxygen reactive species (ROS) (e.g., H_2_O_2_) in the cell wall is necessary to promote the auxin-induced growth of coleoptile sections and that the removal of reactive oxygen species (ROS) inhibited elongation growth.

The toxic effects of heavy metals such as Pb or Cd induce oxidative stress in plants, which is associated with the production of ROS [15,21,22]. Therefore, it was hypothesized that the hormetic effect on the growth of the coleoptile sections excised from seedlings that had been treated with Pb that was observed by Małkowski et al. [20] was associated with the induction of oxidative stress in the seedlings and the accumulation of ROS (e.g., H_2_O_2_). The coleoptile sections contained higher concentrations of ROS, and therefore, after the exogenous IAA was administered, showed a higher elongation growth (hormesis). To verify this hypothesis, the main goal of the study was to determine:

– whether the hormetic effect of elongation growth of the coleoptile sections cut from corn seedlings that had previously been treated with Pb that was observed by Małkowski et al. [20] would also occur in the case of Cd-treated plants

– whether stimulating the elongation growth of coleoptile sections is correlated with higher H_2_O_2_ and IAA content in the sections

– whether the relationships between stimulating growth (hormesis) and the content of H_2_O_2_ and/or IAA that was observed during studies conducted with coleoptile sections will be confirmed in studies with whole corn seedlings

– whether the changes that were observed in the seedling shoots are related to changes in photosynthesis and transpiration rates

## 2. Results

### 2.1. Experiment with the Coleoptile Sections

#### 2.1.1. Influence of Cd and Pb on the Elongation Growth of Corn Coleoptile Sections

**Figure 1:**
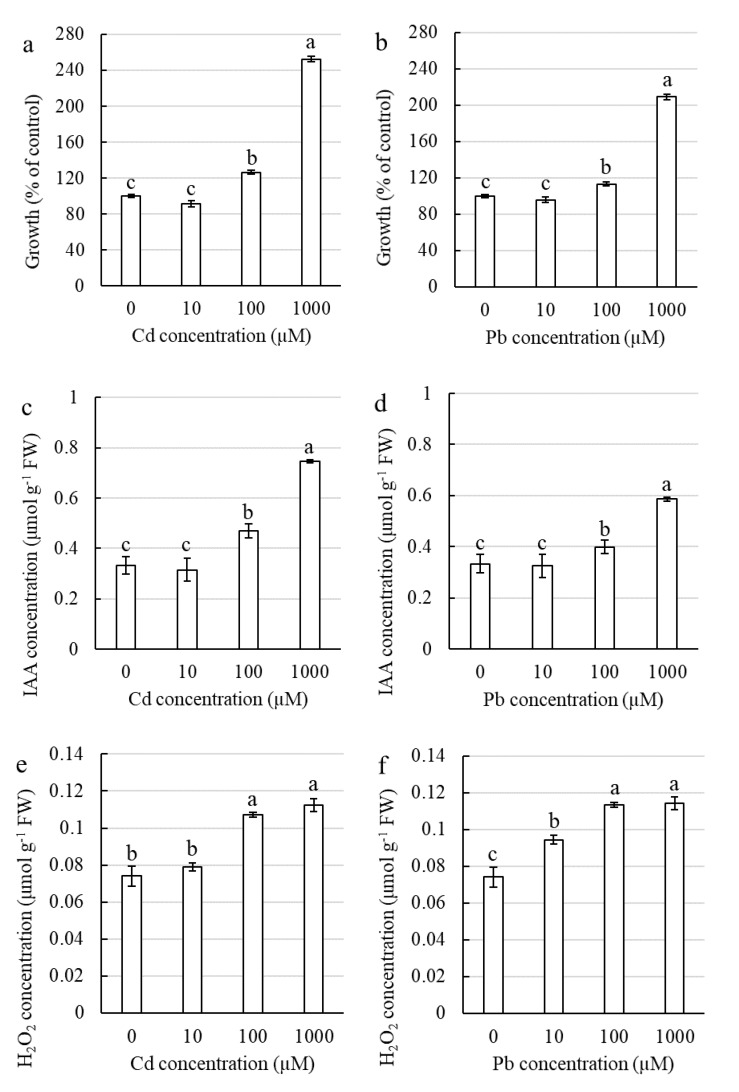
Physiological characteristics of the corn coleoptile sections that had been excised from the seedlings that had been treated with Cd or Pb for 24 h. The growth of sections was measured after incubation in APW for 24 h—(**a**) Cd and (**b**) Pb; concentration of IAA—(**c**) Cd and (**d**) Pb; concentration of H_2_O_2_—(**e**) Cd and (**f**) Pb. The values are the means ± SE (*n* = 3). Means followed by the same letter are not significantly different from each other using the LSD test (*p* < 0.05).

A significant stimulation of elongation growth was observed in the coleoptile sections that had been excised from the corn seedlings that had been treated with 1000 µM Cd or Pb and incubated for 24 h in the control medium (APW) without heavy metals. As a result, there was a three-fold and two-fold increase in elongation growth for the Cd- and Pb-treated plants, respectively, compared to the control. The stimulation of the growth of the sections that had been excised from the seedlings that had been treated with 100 µM Cd or Pb was lower compared to 1000 µM, but it was still significantly higher than in the control (Figure 1a,b).

#### 2.1.2. Influence of Cd and Pb on the Auxin Concentration in Corn Coleoptile Sections

In the coleoptile sections that had been excised from the control seedlings, the auxin concentration was approximately 0.33 µmol g^−1^ FW (Figure 1c,d). A significant increase in the auxin concentration was observed in the coleoptile sections that had been excised from the seedlings growing for 24 h in the medium with 1000 µM Cd or Pb. In the plants that had been treated with Cd, the auxin content in the coleoptile sections was more than two-fold higher (124%) (Figure 1c), whereas in the sections that had been excised from the Pb-treated plants, it was 79% higher (Figure 1d) compared to the control. In the sections that had been excised from the seedlings that had been treated with 100 µM Cd or Pb, the increase in the auxin concentration was still significantly higher compared to the control, 43% and 21% for Cd and Pb, respectively (Figure 1c,d).

#### 2.1.3. Influence of Cd and Pb on the Hydrogen Peroxide Concentration in Corn Coleoptile Sections

The H_2_O_2_ concentration in the coleoptile sections that had been excised from the control seedlings was 0.07 µmol g^−1^ FW (Figure 1e,f). The concentration of 10 µM did not result in significant changes in the H_2_O_2_ concentration (for Cd) (Figure 1e) or increased concentration of hydrogen peroxide in coleoptile sections slightly (37% for Pb) (Figure 1f). While treating the seedlings with Cd or Pb at concentrations of 100 and 1000 µM considerably increased the H_2_O_2_ content in the excised coleoptile sections by 57% compared to the control (Figure 1e,f).

### 2.2. Experiment with the Corn Seedlings

The experiments were conducted on 14-day-old seedlings that had been treated with Cd or Pb for the last four days.

#### 2.2.1. Influence of Cd and Pb on the Elongation Growth of Corn Shoots

**Figure 2:**
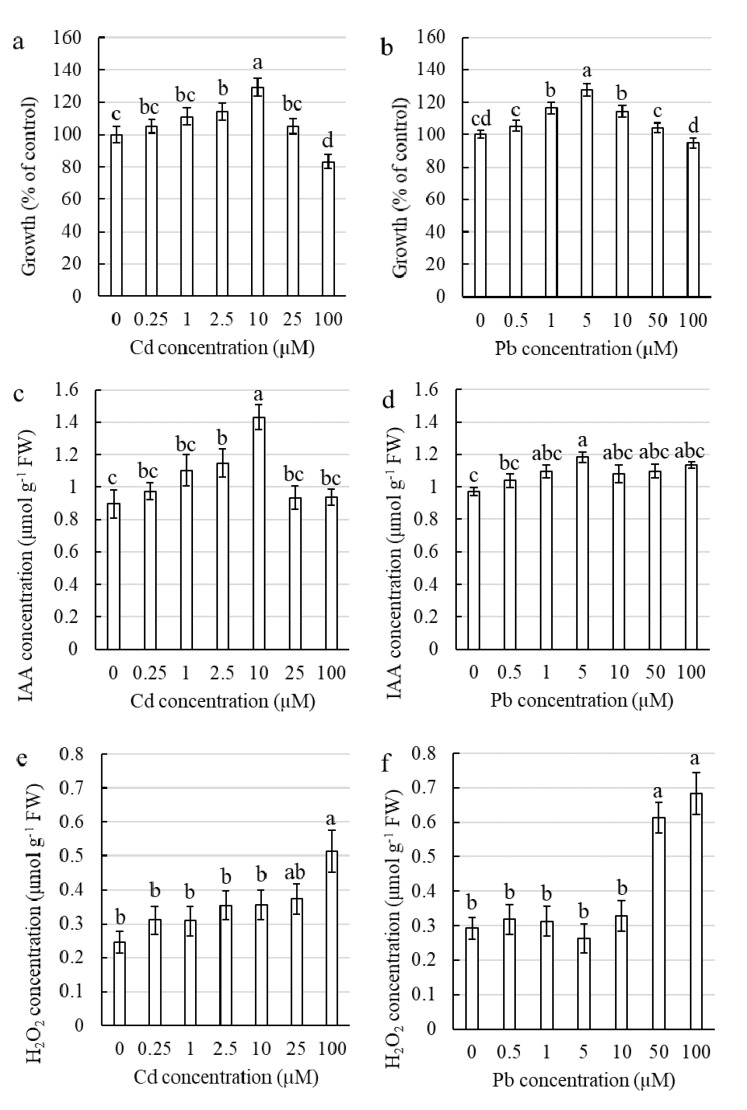
Physiological characteristics of the corn shoots treated with Cd or Pb for four days. Growth of shoots—(**a**) Cd, (**b**) Pb; Auxin concentration in the leaves—(**c**) Cd, (**d**) Pb; H_2_O_2_ concentration in the leaves—(**e**) Cd, (**f**) Pb. The values are the means ± SE (*n* = 8, except for growth where *n* = 18). Means followed by the same letter are not significantly different from each other using the LSD test (*p* < 0.05).

In the seedlings that were treated with Cd for four days, a statistically significant stimulation of growth was observed only at concentrations of 2.5 and 10 µM (Figure 2a). A significant inhibition of growth was measured for the plants treated with Cd at a concentration of 100 µM compared to the control (Figure 2a). A significant stimulation of growth of corn shoots treated with Pb was observed for concentrations of 1, 5 and 10 µM, although there was significant inhibition for the highest tested heavy metal (HM) concentration (Figure 2b). It is worth noting that the highest level of growth stimulation was measured for concentrations of 10 µM and 5 µM of Cd and Pb, respectively (Figure 2a,b).

#### 2.2.2. Influence of Cd and Pb on Auxin and Hydrogen Peroxide in Corn Leaves

The highest and statistically significant (compared to the control) content of auxin was measured for corn leaves in plants treated with Cd at the concentration of 2.5 and 10 µM (Figure 2c). In the plants that were treated with Pb at a concentration of 5 µM, there was a considerably higher content of IAA compared to the control, which was the only significant difference among all of the tested Pb concentrations (Figure 2d).

In the plants treated with Pb, there was a statistically significant increase in H_2_O_2_ content in the leaves for the 50 and 100 µM concentrations, whereas in the Cd-treated plants, there was a considerably higher content of H_2_O_2_ compared to the control only for a concentration of 100 µM of Cd (Figure 2e,f).

#### 2.2.3. The Toxic Effect of Cd and Pb on the Photosynthetic Apparatus

**Figure 3:**
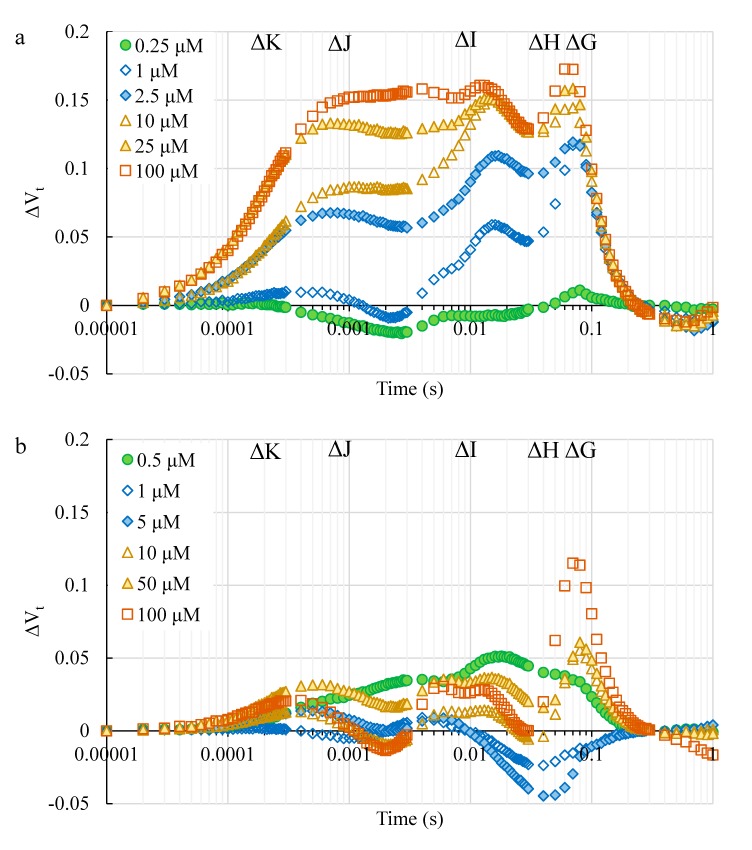
The effect of Cd (**a**) and Pb (**b**) on the relative variable fluorescence of the chlorophyll *a* (ΔV_t_= ((F_t_ – F_0_)/F_v_) – V_control_) of the studied corn leaves. For the ΔV_t_ analysis, the fluorescence of the leaves of the control plant was the reference and equaled 0. The values are the means (*n* = 15).

To highlight the toxic effect of Cd and Pb on photosystem II, the curves of the relative variable fluorescence (∆V_t_) were plotted (Figure 3). ΔV_t_ was calculated as the subtraction between the fluorescence curves that registered for the plants treated with the specific concentrations of HM and the averaged fluorescence values from the control plants (ΔV_t_ = ((F_t_ – F_0_)/F_v_) – V_control_). The toxic effect of Cd was clearly visible in the time-course of the ΔVt curves. Only for a concentration of 0.25 µM Cd was the ΔVt curve comparable to the control (Figure 3a). At low concentrations, Cd seemed to mainly inhibit the activity of the FNR (Ferredoxin-NADP+ Reductase) complex (high ∆H and ∆G steps). As the concentration of Cd was increased, the characteristic peaks that might indicate damage to the individual elements of the electron-transport chain, such as the oxygen evolving complex (Figure 3a), started to appear (∆K, ∆J and ∆I).

The plants treated with Pb were characterized by a much milder time-course of the ΔV_t_ curves (Figure 3b). The greatest changes were observed for the ∆H and G step, which could be correlated with damage to the FNR. The concentrations of 1 µM and 5 µM of Pb resulted in ∆H peaks that were below the control, which may indicate a higher efficiency of the final electron acceptors in PSI compared to the control (Figure 3b). It is noteworthy that the hormetic effect of the growth of the seedlings treated with Cd was found simultaneously with a significantly high decrease in the performance of the photosystems, whereas the activity of the photosynthetic apparatus of the Pb hormetic plants was comparable to the control.

**Figure 4:**
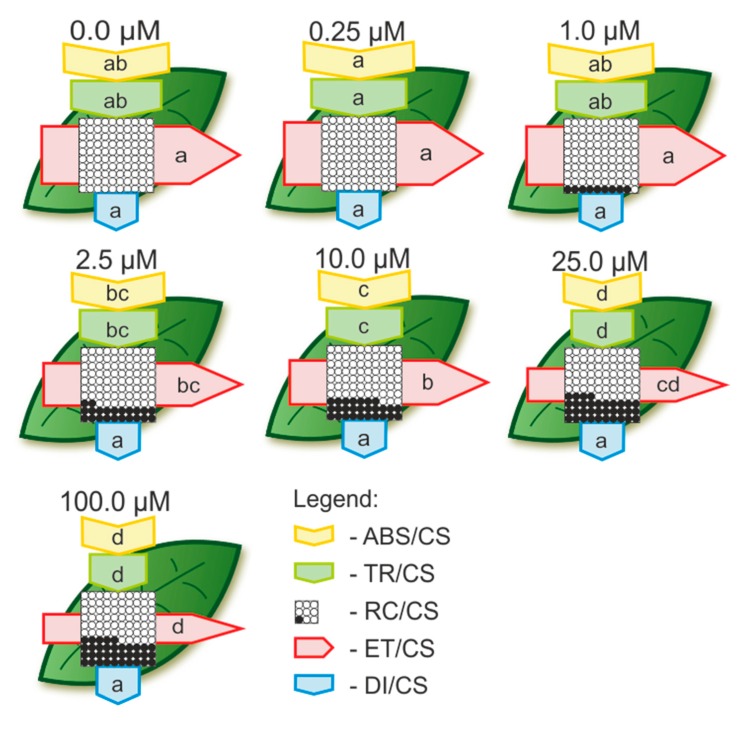
Leaf models showing the phenomenological energy fluxes per the excited cross sections (CS) of corn leaves treated with different Cd concentrations. Each relative value is the mean (*n* = 15) and is represented by the size of the correct parameters (arrows). Letters in the arrows correspond to the statistical significance (LSD test, *p* < 0.05). ABS/CS—absorption flux per CS approximated; TR/CS—trapped energy flux per CS; ET/CS—electron transport flux per CS; RC/CS—% of active/inactive reaction centers as circles inscribed in the square (white = active, black = inactive); DI/CS—dissipated energy flux per CS.

**Figure 5:**
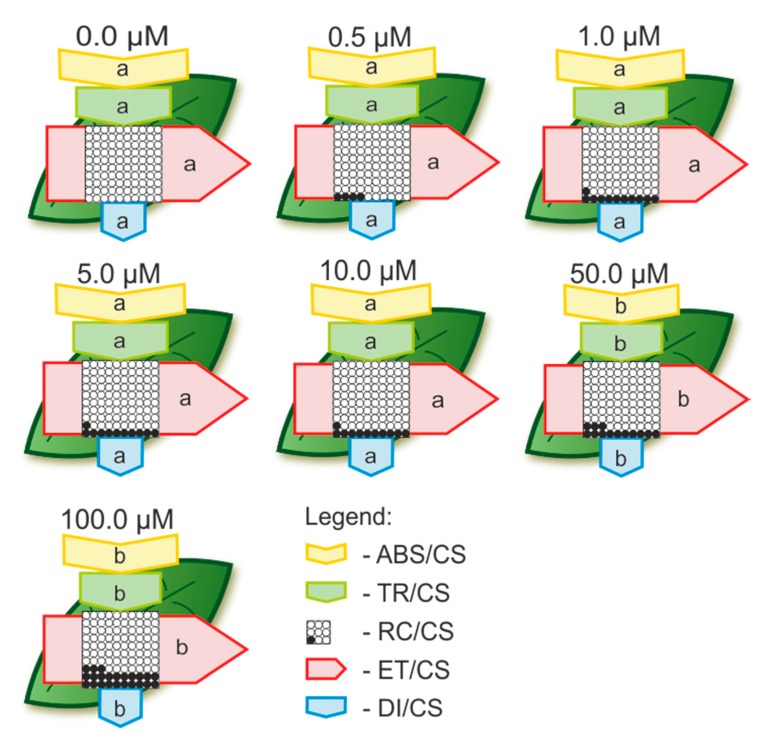
Leaf models showing the phenomenological the energy fluxes per excited cross sections (CS) of the corn leaves treated with different Pb concentrations. Each relative value is the mean (*n* = 15) and is represented by the size of the correct parameters (arrows). Letters in the arrows correspond to the statistical significance (LSD test, *p* < 0.05). ABS/CS—absorption flux per CS approximated; TR/CS—trapped energy flux per CS; ET/CS—electron transport flux per CS; RC/CS—% of active/inactive reaction centers as circles inscribed in the square (white = active, black = inactive); DI/CS—dissipated energy flux per CS.

The phenomenological pipeline models of energy fluxes through the leaf cross sections of the corn plants treated with different concentrations of Cd or Pb are presented in Figure 4 (Cd) and Figure 5 (Pb). The plants treated with Cd at a concentration of 0.25 µM were characterized by the highest values of the parameters that described all of the fluxes, but they did not differ significantly compared to the control (Figure 4). With an increase in the Cd concentration in the medium, there was an increase in the toxic effect of this metal on photosynthetic apparatus. At a concentration of 25 µM Cd, the electron transport through PSII was inhibited to 57% of the control and for 100 µM of Cd, it was inhibited to 50% of the control values. The percentage of active reaction centers in 100 µM of Cd decreased to 65% of the control value (Figure 4).

Treatment with Cd had no impact on dissipated energy through the cross sections of the leaves. A significant inhibition of energy flux parameters in plants treated with Pb was only observed at concentrations of 50 and 100 µM (Figure 5). These concentrations also caused a decrease in the dissipated energy through cross section. The limitation of electron transport at a concentration of 100 µM of Pb was also observed, but only by 10% compared to the control. The inhibition of reaction center activity at this concentration was determined to be 77% of the control (Figure 5).

**Figure 6:**
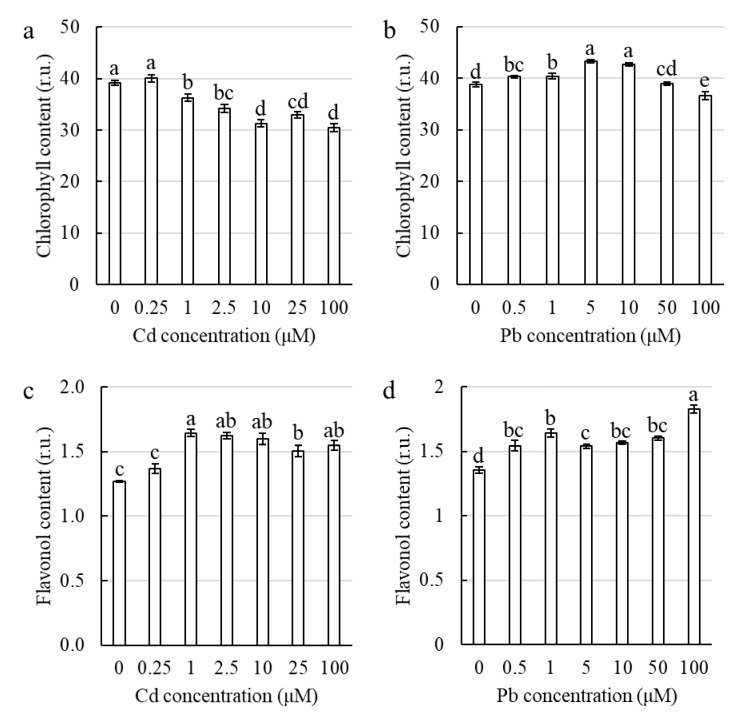
The effect of Cd and Pb on the chlorophyll and flavonol content in the corn leaves. Cd (**a**,**c**) and Pb (**b**,**d**). The values are the means ± SE (*n* = 15). Means followed by the same letter are not significantly different from each other using the LSD test (*p* < 0.05).

Treatment with Cd caused a decrease in the chlorophyll content in the corn leaves (Figure 6a). As a result, the chlorophyll content varied from 75% to 80% of the control for the concentrations from 10 µM to 100 µM of Cd. In contrast to Cd, most of the investigated Pb concentrations caused a significant increase in the chlorophyll content in the corn leaves (Figure 6b). For both of the investigated heavy metals, there was an increase in the flavonol content in the leaves with an increase in metal concentration in the hydroponic medium (Figure 6c,d). It is noteworthy that the higher content of flavonols in the leaves was related with a hormetic increase in growth for both metals, whereas an increase of the chlorophyll content at a concentration that caused hormesis was only observed for the corn treated with Pb.

**Figure 7:**
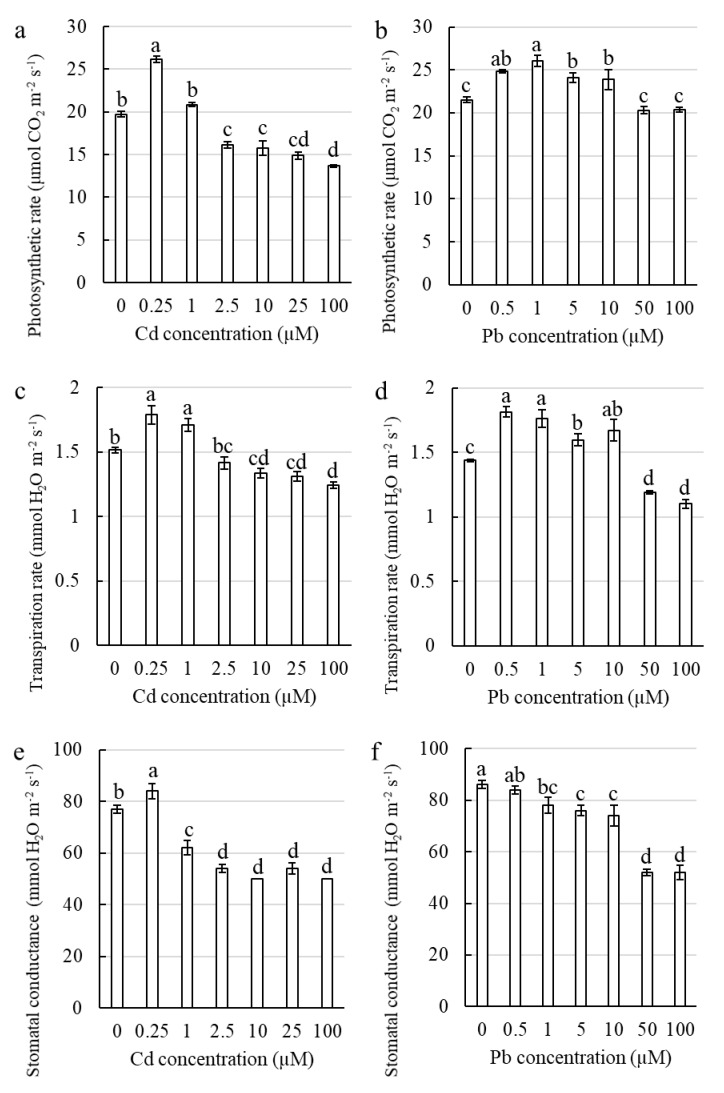
The effect of Cd and Pb on the photosynthetic and transpiration rate and stomatal conductance in the corn leaves. Cd (**a**,**c**,**e**) and Pb (**b**,**d**,**f**). The values are the means ± SE (*n* = 15). Means followed by the same letter are not significantly different from each other using LSD test (*p* < 0.05).

There was a stimulation of the photosynthetic rate by Cd only at a concentration of 0.25 μM, whereas at concentrations from 2.5 μM to 100 μM of Cd, there was a significant decrease compared to the control (Figure 7a). At the lowest investigated concentration, Cd considerably stimulated the transpiration rate and stomatal conductance, but the higher concentrations decreased these parameters compared to the control (Figure 7c,d). It is worth noting that the increase in growth of the corn shoots was measured at the concentrations of Cd at which all of the gas exchange parameters were significantly lower compared to the control.

In contrast to Cd, there was a significant stimulation of the photosynthetic rate for Pb for the range of concentrations from 0.5 to 10 μM, whereas at the highest investigated concentration, the photosynthetic rate did not differ compared to the control (Figure 7b). Moreover, the transpiration rate of corn treated with Pb was characterized by a similar relation as the photosynthetic rate (Figure 7d). No significant increase was only observed for the stomatal conductance compared to the control (Figure 7e). Interestingly, there was an increase in the photosynthetic and transpiration rate that was caused by the Pb treatment for the concentrations at which an increase in the growth of the corn shoots was also documented.

On the basis of the principal component analysis (PCA), the positive correlation between the auxin concentration and hormetic growth stimulation by both HMs was demonstrated (Figure S1, Tables S1 and S2). PCA made it possible to isolate each concentration as a separate group characterized by unique plant physiological status. What is particularly noteworthy is that plants showed hormetic growth stimulation formed groups apart from both control and high concentrations of both heavy metals (Figure S1).

## 3. Discussion

It has been well documented that incubating corn coleoptile sections in a medium with Pb or Cd inhibits the elongation growth of the sections [26,27,28,29]. In our preliminary study, however, the corn coleoptile sections that had been excised from seedlings that had been treated with Pb and incubated in a medium without Pb grew substantially better than the coleoptile sections that had been excised from the control seedlings [20]. In the current study, we showed that stimulating the growth of coleoptile sections by pretreating the seedlings with Pb or Cd is the result of the higher content of IAA and H_2_O_2_ in these sections (Figure 1). This finding is in agreement with the data presented by Schopfer et al. [32], who documented that the presence of oxygen reactive species (ROS) (e.g., H_2_O_2_) in the cell wall is necessary to promote the auxin-induced growth of coleoptile sections. Most investigations have shown that treating plants with heavy metals decreases the content of IAA in plants, see [33] and literature therein [34,35,36,37,38,39]. In the current study, we found that Pb and Cd increased the content of IAA in the corn coleoptile sections, but only at the concentrations at which stimulation of elongation growth was observed (Figure 1). Hence, we can conclude that for an increase in IAA and H_2_O_2_ content is necessary for the elongation growth stimulation by HMs.

Because as a model object, coleoptile sections have a simple structure and are a short-lived organ [40], we conducted other experiments with corn seedlings. The main goal of the experiments with the seedlings was to determine whether an increase in both the IAA and H_2_O_2_ content in the shoots is necessary to induce the hormetic effect on growth.

Many investigations have been conducted to study the effects of heavy metal toxicity on plants. However, fewer works have addressed the stimulatory effect of sub-toxic levels of HMs—the phenomenon of hormesis. However, hormesis has been gaining more and more interest in recent years [2,3,4,41]. The stimulatory effect of low concentrations of Cd on plant growth was reported for *Lonicera japonica* by Jia et al. [8,42] and for *Brassica juncea* by Seth et al. [43]. Moreover, it was reported that Pb also had a stimulatory effect on the growth of corn [44] and *Arabis paniculata* [45]. In our research, we observed a significant increase in the growth of the shoots of the corn by 29% and 27%, respectively, compared to the control when treated with 10 µM Cd and 5 µM Pb (Figure 2a,b).

The majority of the experiments that have been conducted that have focused on the effect of Cd and Pb on plants have reported an increase in the reactive oxygen species (ROS) level as a response to HM treatment that was accompanied by an inhibition of growth [46,47,48,49,50,51,52]. However, in our research, which was conducted on corn shoots treated with Cd or Pb at the concentrations for which growth stimulation was found, there was no significant change in the H_2_O_2_ content compared to the control (Figure 2e,f). Lin et al. [53] and Jia et al. [8] also showed no significant increase in the level of oxidative stress in plants in which growth stimulation was observed under a low Cd treatment compared to the untreated plants. These results contradict the results that were obtained in the current study for coleoptile sections for which an increase in the H_2_O_2_ content was always necessary to induce hormesis (Figure 1).

Phytohormones such as auxins have been found to play an important role in plant tolerance and the alleviation of the stress that is induced by HMs [54]. It has been suggested that the cellular level of auxins increases under a low level of abiotic stress, which stimulates vegetative growth, thus leading to the hormetic effect [3,55]. Although Elobeid and Polle ([33] and literature therein) reported that a high concentration of Cd in *Glycine max* inhibited auxin biosynthesis and reduced growth, whereas exposure to a low concentration of Cd stimulated auxin biosynthesis. Other studies have shown that the application of exogenous auxin improves a plant’s protection against HM toxicity and can reverse the growth inhibition that is caused by heavy metals [54]. Liphadzi et al. [56] documented that the addition of exogenous IAA caused a significant increase in the biomass of the roots and stems of *Helianthus annuus* plants grown in soil that had been moderately contaminated with Pb compared to the untreated plants. We found that treatment with Cd and Pb at a concentration of 10 µM and 5 µM, respectively, at which we observed a hormetic effect, significantly increased the content of IAA in the corn shoots compared to the untreated plants (Figure 2c,d). The same trend was observed in our experiments with the coleoptile sections in which the induction of elongation growth was always connected with a higher IAA content (Figure 1). Based on our results, we suggest that the increase in auxin content in response to subtoxic levels of HMs such as Cd and Pb plays a key role in the phenomenon of hormesis in plants.

Figlioli et al. [44] found that all of the investigated concentrations of Pb (from 10 μM to 1000 μM) significantly stimulated the growth (both the length of the seedlings and their fresh weight) of corn plants in cultivated garden soil. However, the maximum quantum efficiency of PSII did not differ significantly, whereas the chlorophyll content was significantly higher compared to the control, but only for plants treated with 1000 μM of Pb [44]. A similar effect of Pb was observed on *Pisum sativum* by Rodriguez et al. [57] and on corn by Nyitrai et al. [58]. An increase in the chlorophyll content that was caused by Pb was also confirmed in *Populus* × *canescenc* trees [59] and *Arabis paniculata* [45]. The results presented in the current study also confirmed that the phenomenon of hormesis, which is caused by Pb, was positively correlated with a higher chlorophyll content and that there was no significant difference in the PSII yield compared to the control (Figure 3b, Figure 5 and Figure 6b). On the other hand, improvements in the content of photosynthetic and accessory pigments (such as chlorophyll a, chlorophyll b, total chlorophyll and/or carotenoids) were observed in the leaves of different plant species after Cd exposure [4]. Conversely, González et al. [11] observed a decrease in the chlorophyll content and PSII performance in barley plants, which simultaneously showed an increase in growth that was caused by Cd treatment. Generally, the stimulation of growth by Cd is positively correlated with a significant decrease in the total chlorophyll content and activity of the photosystems [11,43]. The results presented in this paper also confirmed this relationship, which may additionally prove the high toxic effect of Cd on these parameters (Figure 3a, Figure 4 and Figure 6a).

There is a dearth of data on the effect of both Cd and Pb on the content of flavonols during hormesis. However, Zhang et al. [60] found that the addition of exogenous flavonols diminished the toxic effect of Pb on growth and the ROS level in *Arabidopsis thaliana*. They also discovered that flavonols increased the activities of the antioxidant enzymes and detoxified the ROS, which prevented oxidative damage [60]. Moreover, Cetin et al. [61] described the dose-dependent stimulation of the synthesis of flavonols in grape cell suspension cultures that had been treated with Cd. These results are in agreement with the data obtained in our work, which suggests that the corn plants maintained the H_2_O_2_ content at the level of the control due to a higher content of flavonols, which seems to be a part of the hormesis mechanism (Figure 6c,d). In this work, we showed that corn treated with Cd or Pb and that was characterized by hormetic growth stimulation also had a significant increase in the flavonol content in the leaves for both heavy metal treatments for the first time.

There are many papers that discuss the toxic effect of Cd or Pb on the gas exchange in plants [4,62,63,64,65,66,67], but there is a paucity of published works that describe the gas exchange parameters in relation to the hormetic stimulation of growth. Ban et al. [68] showed that a low dose of Pb stimulated growth, the photosynthetic rate, transpiration and stomatal conductance in corn. In the shoots of many plant species, the hormesis that is caused by Cd is associated with an increase in the photosynthesis activity, which improves plant development [4,41]. In our study, the hormetic stimulation of growth that was caused by Pb was correlated with increased photosynthetic and transpiration rates, while the hormetic stimulation of growth that was caused by Cd led to a significant decrease of all of the investigated plant gas exchange parameters (Figure 7). Based on these results, we can suggest that stimulating growth by HMs during hormesis does not have to be correlated with an increase in CO_2_ assimilation and transpiration.

**Figure 8:**
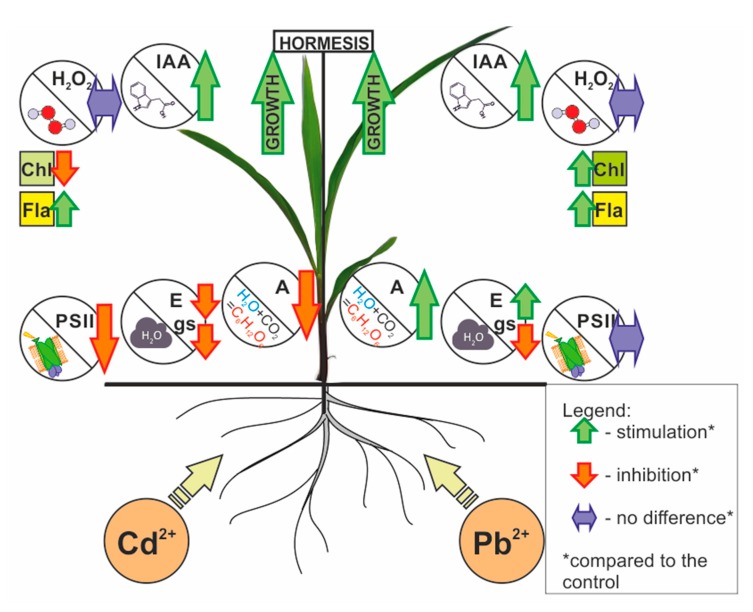
Model summarizing the differences in the corn plant response during hormetic growth stimulation after treatment with low doses of Pb or Cd. It was documented that the shoot growth stimulation for both metals was correlated with an increase in the IAA and flavonol content in the shoots. This increase in the content of both substances was accompanied by the maintenance of the H_2_O_2_ content at the level of the control. In conclusion, an increase in the IAA content and a lack of oxidative stress seem to play a key role in the hormetic stimulation of shoot growth by Pb and Cd. Abbreviation list: A—photosynthetic rate; Chl—chlorophyll content; E—transpiration rate; Fla—flavonol content; gs—stomatal conductance; H_2_O_2_—hydrogen peroxide content; IAA—auxin content; PSII—photosystem II performance and quantum yield.

To summarize, we confirmed that hormesis is a complex process, which is still not fully understood or investigated. In this work, we present the effect of two heavy metals (Pb and Cd), which are characterized by different physicochemical properties, on the hormetic stimulation of corn shoot growth. Based on our results, we conclude that there are common features that seem to be necessary to induce the hormetic stimulation of shoot growth by heavy metals. They are an increase in the auxin and flavonol content and the maintenance of hydrogen peroxide at the level of the control (Figure 8). We would like to stress that an increase in the IAA and flavonol content have been proposed as the key factors in hormesis here for the first time. In general, the literature on metals and hormesis is dominated by studies on the role of oxidative stress and phytohormones such as abscisic acid, ethylene or jasmonates, with little information on the role of IAA and flavonols [3,4,6,41,54]. The results presented here also suggest that an increase in the photosynthesis and transpiration rates are not necessary for stimulating hormetic growth. To sum up, the presented results shed new light on the mechanism of hormesis and can open new research directions, which should encompass analyses of the gene expression and phytohormone crosstalk.

## 4. Materials and Methods

### 4.1. Plant Material

In the experiment with coleoptiles sections the caryopsis of *Zea mays* L. cv. ‘KOSMO 230′ were used. First, they were soaked in tap water for 2 h; then, the seeds were sown in plastic trays that had been lined with moistened cellulose sheets. The trays were placed in an incubator (MIR-533, SANYO, Moriguchi, Japan) and left for three days in the dark for seed germination at a temperature of 27 ± 0.5 °C, ≈100% humidity and were watered as needed.

The caryopsis of cv. ‘LOKATA’ were used for the experiment with corn seedlings, because the seeds of cv. ‘KOSMO 230′ were no longer available. A preliminary study showed that the coleoptile sections that had been excised from the seedlings of cv. ‘LOKATA’ that had been treated with Pb or Cd reacted in a way that was similar to the sections that had been excised from the seedlings of cv. ‘KOSMO 230′. As a result, the caryopsis of cv. ‘LOKATA’ were germinated in the same manner as the cv. ‘KOSMO 230′ seeds.

### 4.2. Hydroponic Cultures

In the experiment with coleoptiles, the three-day-old etiolated *Z. mays* L. cv. ‘KOSMO 230′ seedlings with equal coleoptile lengths (1–1.5 cm) were transferred into hydroponic cultures (100 seedlings/2.5 l) with artificial pond water (APW) composed of the following: 1 mM KCl, 0.1 mM NaCl and 0.1 mM CaCl_2_ dissolved in deionized water [14]. The initial pH of APW was established at 5.8 ± 0.1. The following treatments were applied APW (control), APW + 10 µM, APW + 100 µM and APW + 1000 µM of CdCl_2_ or PbCl_2_. The seedlings were cultivated for the next 24 h in the incubator in conditions similar to those described in 4.1. From four-day-old etiolated corn seedlings, 1 cm long coleoptile sections were excised with blade razor starting 3 mm from the tip; next, the first leaf was removed using a dissecting needle. The concentrations of H_2_O_2_ and auxin was measured in the prepared sections, or they were incubated for 24 h in the APW for the measurements of elongation growth.

In the experiment with seedlings, the three-day-old etiolated corn seedlings cv. ‘Lokata’ with only the primary root and coleoptile length of 3.5 ± 0.5 cm were transferred into hydroponic cultures, which were carried out in plastic containers (nine seedlings/container) that were filled with 2850 mL (315 mL/seedling) of a nutrient solution. A Hoagland solution [69] was used in the hydroponic cultures with the initial pH established at 5.9 ± 0.05. The seedlings were grown in a greenhouse under artificial light using sodium lamps (HPS), a photoperiod of 16/8 h day/night and an average energy of light of 250 μmoL E m^−2^ s^−1^. The temperature in the greenhouse was 20 ± 1 °C and the air humidity was 30 ± 5%. During the first seven days of the experiment, medium was changed twice. After seven days of plant cultivation the medium was changed into a Hoagland solution (control) or a Hoagland solution with 0.25, 1, 2.5, 10, 25 or 100 μM CdCl_2_ or 0.5, 1, 5, 10, 50 or 100 μM of PbCl_2_. The concentrations of the metals were selected based on preliminary studies.

### 4.3. Growth Measurement

In the experiment with coleoptiles, a column of 10 coleoptile sections was placed on a stainless-steel wire to preserve their natural vertical orientation, and the length of the column was measured with an accuracy to 0.1 mm using calipers. Next, the column was introduced into a measuring cylinder that had been filled with 100 mL APW (10 mL/segment; this volume prevents the medium pH to be changed by the coleoptile sections) with an initial pH 5.8–6.0 and incubated in the dark for the next 24 h at room temperature. After 24 h, the length of the column was measured once again. The endogenous growth of the coleoptile sections was assessed as the difference between the length of the sections of a column after 24 h of incubation and the length of the sections of a column at the start of the experiment. The measurements were repeated twice.

In the experiment with corn seedlings, the length of the shoots was measured after seven days of cultivation on control solution by measuring from the first node to the end of the longest leaf. Then, after the four days of growth in the medium with HMs, the length of the shoots was measured again. The growth of the shoots was assessed as the difference between the length of the shoots before and the length of shoots after four days of metal treatment. From each container, the smallest seedling was rejected and the remaining eight seedlings were used for the other measurements. Because Pb easily precipitates in the presence of phosphate ions, 2 mM of NH_4_H_2_PO_4_ were not administrated to the medium with PbCl_2_ [17,18,70,71]. All of the investigated physiological parameters were measured on the fourth day of the HM treatment on second fully developed leaf counting from the bottom of the seedling.

### 4.4. Measurement of the Hydrogen Peroxide and Indole Compound Content in the Corn Coleoptile Sections and Leaves

The hydrogen peroxide content in the corn coleoptile sections and leaves was measured according to the method of Bouazizi et al. [72]. Fresh leaf tissues (150 mg) were homogenized in 1.5 mL of 0.1% trichloroacetic acid (TCA). The homogenate was centrifuged at 12,000× *g* for 15 min and 0.5 mL of the supernatant was added to a 0.5 mL potassium phosphate buffer (10 mM, pH 7.0) and 1 mL potassium iodide (KI) (1 M). The absorbance was measured at 390 nm and the content of H_2_O_2_ was determined using a standard curve.

The content of the indole compounds in the corn coleoptile sections and leaves was measured according to the method of Wójcikowska et al. [73]. Fresh leaf tissues (150 mg) were homogenized in 1.5 mL of 10 × PBS (phosphate buffer solution). The homogenate was centrifuged at 15,000× *g* for 25 min and 0.3 mL of the supernatant was added to 0.025 mL of orthophosphate acid (0.01 M) and 1.2 mL of Salkowski’s reagent (150 mL H_2_SO_4_; 250 mL ddH_2_O; 7.5 mL 0.5 M FeCl_3_ × 6H_2_O). The absorbance was measured at 530 nm and the content of indole compound was determined using a standard curve. It should be emphasized that Wójcikowska et al. [73] showed a direct correlation between the content of the indole compounds and indolyl-3-acetic acid (IAA) in plant tissue, and therefore, the term auxin (IAA) content is used in the current study. A spectrophotometer (SPECORD^®^ 250, Analytik Jena, Jena, Germany) was used to determine the hydrogen peroxide and auxin content in the plant samples.

### 4.5. Measurements of the Photosynthetic Characteristics, Transpiration and Pigment Content

All of the measurements were performed on the second fully developed leaf counting from the bottom of the seedling for eight plants from each treatment after four days of HM treatment. The chlorophyll *a* fluorescence (ChlF) was measured using a Plant Efficiency Analyzer (PocketPEA fluorimeter, Hansatech Instruments Ltd., King’s Lynn, England). Before the measurement, each selected leaf was adapted in the dark for 30 min using dedicated leaf clips. After adaptation, a saturating light pulse of 3500 μmol photons m^−2^ s^−1^ was applied for 1 s, which closed all of the reaction centers, and then the fluorescence parameters were measured. The measurements were performed without damaging the plant material.

The plant gas exchange parameters such as the net photosynthetic rate (A), stomatal conductance (gs) and transpiration rate (E) were measured on the same leaves as the ChlF. The measurements were performed at the end of experiment using an infrared gas analyzer with a special narrow chamber (LCpro+, ADC Bioscientific, Hoddesdon, UK) under controlled climate conditions (T = 21 °C, PAR = 1500 μmol m^−2^ s^−1^). The measurements were performed at noon.

The content of chlorophyll and flavonols was measured using a Dualex sensor (Force-A, Orsay, France). The pigment content was measured on the same leaves as for the ChlF and gas exchange measurements. The measurements were performed without damaging the plant material. The pigment content was calculated on the basis of the fluorescence and light transmission measurement with a 5-mm-diameter probe. More details on pigment content index measurements are presented by Cerovic et al. [74]

### 4.6. Statistical Analysis

The results are shown as the means ± SE. The statistically significant differences among mean values were determined using a one-way ANOVA and the post hoc Fischer LSD test (*p* < 0.05). The statistical analysis was performed using Statistica v.13.1 (Dell Inc., Round Rock, TX, USA). The principal components analysis (PCA) was used to identify the dominant groups of the factors that characterized physiological status of plants treated with Cd or Pb. The pipeline models of the energy fluxes through a leaf’s cross section were done using CorelDRAW X6 (Corel Corp., Ottawa, ON, Canada).

## Supplementary Materials

Supplementary materials can be found at https://www.mdpi.com/1422-0067/21/6/2099/s1.

## Abbreviations

A	photosynthetic rate
ABS/CS	absorption flux per CS
APW	artificial pond water
ChlF	chlorophyll a fluorescence
CS	excited cross section of leaf
DI/CS	dissipation energy flux per CS
ΔVt	control; variable fluorescence
E	transpiration rate
ET/CS	electron transport per CS
FNR	Ferredoxin-NADP+ Reductase
F0	minimal fluorescence, when all PS II RCs are open (at t = 0)
Fm	maximal fluorescence, when all PS II RCs are closed
Fv	maximal variable fluorescence
Ft	fluorescence at time t
gs	stomatal conductance
HM	heavy metal
IAA	auxin
OJIP	transient chlorophyll a fluorescence rise induced during a dark-to-strong light transition, where O is equivalent to F0 and P is for peak equivalent to Fm
PAR	photosynthetic active radiation
PSI	photosystem I
PSII	photosystem II
RC	reaction center of PSII
RC/CS	% of active reaction centers per CS compared to the control
ROS	reactive oxygen species
TR/CS	trapped energy flux per CS
