# Supplementary material for: Hormesis in Plants: The Role of Oxidative Stress, Auxins and Photosynthesis in Corn Treated with Cd or Pb
Source: Int J Mol Sci. 2020 Mar 19;21(6):2099. doi: 10.3390/ijms21062099 (PMC7139973; doi:10.3390/ijms21062099)
Supplement: Supplementary file 1 [file ijms-21-02099-s001.pdf]

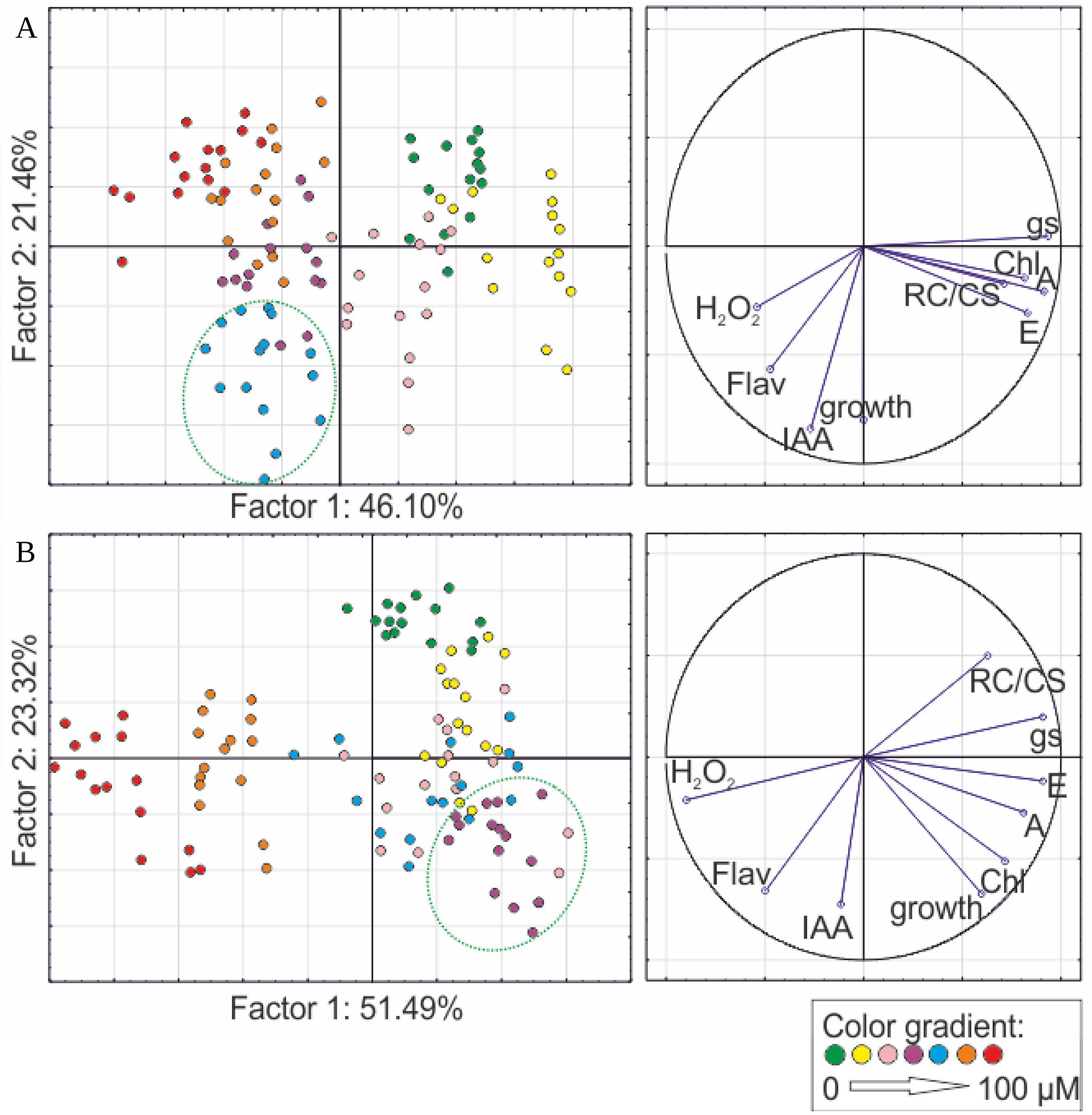

Figure S1. Principal component analysis (PCA) presenting the relationships between selected physiological parameters measured in corn shoots treated with Cd (A) or Pb (B). The green dotted line indicates the concentrations at which hormetic growth stimulation was observed. Abbreviations: A – photosynthetic rate; Chl – chlorophyll content index; E – transpiration rate; Flav – flavonol content index; growth – shoots growth; gs – stomatal conductance; H_2O_2 – hydrogen peroxide concentration; IAA – auxin concentration; RC/CS – percentage of active reaction centers per excited cross section of leaf.

Table S1. Correlation between factors examined in PCA for plants treated with Cd.

	growth	Chl	Fla	E	gs	A	IAA	H_2O_2	RC/CS
growth	1.000000	0.101192	0.330125	0.094444	-0.071728	0.080840	0.560396	-0.028323	0.166741
Chl	0.101192	1.000000	-0.147910	0.630964	0.771425	0.734339	-0.188650	-0.345621	0.475751
Fla	0.330125	-0.147910	1.000000	-0.161816	-0.555539	-0.317600	0.401176	0.286911	-0.232633
E	0.094444	0.630964	-0.161816	1.000000	0.802046	0.881531	0.033929	-0.201500	0.519676
gs	-0.071728	0.771425	-0.555539	0.802046	1.000000	0.862240	-0.247101	-0.316661	0.504085
A	0.080840	0.734339	-0.317600	0.881531	0.862240	1.000000	-0.047210	-0.319834	0.580864
IAA	0.560396	-0.188650	0.401176	0.033929	-0.247101	-0.047210	1.000000	0.454417	-0.011575
H_2O_2	-0.028323	-0.345621	0.286911	-0.201500	-0.316661	-0.319834	0.454417	1.000000	-0.566378
RC/CS	0.166741	0.475751	-0.232633	0.519676	0.504085	0.580864	-0.011575	-0.566378	1.000000

Abbreviations: A – photosynthetic rate; Chl – chlorophyll content index; E – transpiration rate; Flav – flavonol content index; growth – shoots growth; gs – stomatal conductance; H_2O_2 – hydrogen peroxide concentration; IAA – auxin concentration; RC/CS – percentage of active reaction centers per excited cross section of leaf.

Table S2. Correlation between factors examined in PCA for plants treated with Pb.

	growth	Chl	Fla	E	gs	A	IAA	H_2O_2	RC/CS
growth	1.000000	0.867361	0.096756	0.491663	0.326132	0.525343	0.320978	-0.469741	0.072830
Chl	0.867361	1.000000	-0.094176	0.586928	0.486777	0.537779	0.235512	-0.541495	0.270647
Fla	0.096756	-0.094176	1.000000	-0.284877	-0.512818	-0.141167	0.327342	0.578768	-0.634261
E	0.491663	0.586928	-0.284877	1.000000	0.841168	0.917609	-0.040351	-0.730714	0.423573
gs	0.326132	0.486777	-0.512818	0.841168	1.000000	0.741335	-0.229662	-0.811741	0.624591
A	0.525343	0.537779	-0.141167	0.917609	0.741335	1.000000	0.069225	-0.590857	0.241251
IAA	0.320978	0.235512	0.327342	-0.040351	-0.229662	0.069225	1.000000	0.300967	-0.245486
H_2O_2	-0.469741	-0.541495	0.578768	-0.730714	-0.811741	-0.590857	0.300967	1.000000	-0.617828
RC/CS	0.072830	0.270647	-0.634261	0.423573	0.624591	0.241251	-0.245486	-0.617828	1.000000

Abbreviations: A – photosynthetic rate; Chl – chlorophyll content index; E – transpiration rate; Flav – flavonol content index; growth – shoots growth; gs – stomatal conductance; H_2O_2 – hydrogen peroxide concentration; IAA – auxin concentration; RC/CS – percentage of active reaction centers per excited cross section of leaf.
